# Increased expression and altered subcellular distribution of cathepsin B in microglia induce cognitive impairment through oxidative stress and inflammatory response in mice

**DOI:** 10.1111/acel.12856

**Published:** 2018-12-21

**Authors:** Junjun Ni, Zhou Wu, Veronika Stoka, Jie Meng, Yoshinori Hayashi, Christoph Peters, Hong Qing, Vito Turk, Hiroshi Nakanishi

**Affiliations:** ^1^ Department of Aging Science and Pharmacology, Faculty of Dental Science Kyushu University Fukuoka Japan; ^2^ Department of Biochemistry and Molecular and Structural Biology J. Stefan Institute Ljubljana Slovenia; ^3^ Institute für Molekulare Medizin und Zellforshung Albert‐Ludwigs‐Universität Freiburg Freiburg Germany; ^4^ Beijing Key Laboratory of Separation and Analysis in Biomedical and Pharmaceuticals, Department of Biomedical Engineering, School of Life Science Beijing Institute of Technology Beijing China; ^5^ Department of Pharmacology, Faculty of Pharmacy Yasuda Women’s University Hiroshima Japan

**Keywords:** cathepsin B, lysosomal leakage, microglia, mitochondria‐derived reactive oxygen species

## Abstract

During normal aging, innate immunity progresses to a chronic state. However, how oxidative stress and chronic neuroinflammation arise during aging remains unclear. In this study, we found that genetic ablation of cathepsin B (CatB) in mice significantly reduced the generation of reactive oxygen species (ROS) and neuroinflammation and improved cognitive impairment during aging. In cultured microglia, pharmacological inhibition of CatB significantly reduced the generation of mitochondria‐derived ROS and proinflammatory mediators induced by L‐leucyl‐L‐leucine methyl ester (LLOMe), a lysosome‐destabilizing agent. In the CatB‐overexpressing microglia after treatment with LLOMe, which mimicked the aged microglia, CatB leaked in the cytosol is responsible for the degradation of the mitochondrial transcription factor A (TFAM), resulting in the increased generation of mitochondria‐derived ROS and proinflammatory mediators through impaired mtDNA biosynthesis. Furthermore, intralateral ventricle injection of LLOMe‐treated CatB‐overexpressing microglia induced cognitive impairment in middle‐aged mice. These results suggest that the increase and leakage of CatB in microglia during aging are responsible for the increased generation of mitochondria‐derived ROS and proinflammatory mediators, culminating in memory impairment.

## 1 INTRODUCTION

It is widely believed that oxidative stress and inflammation are major causative factors for the progressive decline in motor and cognitive functions that occur during normal aging in humans and animals (Forster et al., 1996; Navarro, Sanchez Del Pino, Gomez, Peralta, & Boveris, 2002). The activation of microglia is the main cellular source of oxidation products and proinflammatory mediators in the brain (Hayashi et al., 2008; Pawate, Shen, Fan, & Bhat, 2004). Cathepsin B (CatB, EC 3.4.22.1), a typical cysteine lysosomal protease, is associated with inflammatory responses by microglia through the production of IL‐1β (Terada et al., 2010). Furthermore, CatB is a potential molecular switch that shifts microglia/macrophages toward the neurotoxic phenotype through autophagic activation of nuclear factor‐κB (NF‐κB; Ni et al., 2015). More recently, CatB has been demonstrated to play a critical role in neuroinflammation and impairment of learning and memory induced by chronic systemic exposure to lipopolysaccharide derived from *Porphyromonas gingivalis*, the major periodontal bacteria, in middle‐aged mice (Wu et al., 2017).

Recent publications have indicated that mitochondria‐derived reactive oxygen species (ROS) act as signaling molecules triggering the production of proinflammatory mediators (Nakahira et al., 2011). Mitochondrial DNA (mtDNA) is highly susceptible to the damage produced by ROS because of its close proximity to ROS generated through the respiratory chain and the paucity of protective histones (Hayashi et al., 2008; Kanki et al., 2004). The accumulation of mtDNA damage during aging leads to dysfunction of the respiratory chain, especially complex I and IV, resulting in enhanced ROS production and culminating in age‐dependent cognitive impairment (Corral‐Debrinski et al., 1992; Lin, Simon, Ahn, Kim, & Beal, 2002). Besides regulation of the copy number, the mitochondrial transcription factor A (TFAM) is closely associated with stabilization of mtDNA structures (Kanki et al., 2004). The overexpression of human TFAM in mice significantly inhibits age‐dependent accumulation of mtDNA damage in microglia, resulting in a decrease in the production of ROS and the subsequent activation of NF‐κB‐mediated inflammatory responses and thereby leading to the improvement of age‐dependent motor and memory decline (Hayashi et al., 2008; Nakanishi & Wu, 2009). It is interesting to note that the overexpression of TFAM and genetic depletion of CatB exhibit similar cellular and behavioral phenotypes in mice during aging, including improvement of excessive neuroinflammatory responses, oxidative stress, and memory decline (Hayashi et al., 2008; Wu et al., 2017).

These observations may lead to a deduction that CatB could induce subnormal levels of TFAM during aging, resulting in impairment of learning and memory through excessive oxidative and inflammatory responses. Lon, the major ATP‐dependent protease in the mitochondrial matrix, regulates mtDNA transcription by the selective degradation of TFAM (Matsushima, Goto, & Kaguni, 2010). Therefore, it is likely to speculate that pre‐TFAM synthesized in the cytosol may be a potential target substrate for CatB leaked in the cytosol of microglia during aging. In the present study, we have provided evidence that CatB enhances oxidative stress and inflammatory response in microglia by proteolytic degradation of TFAM after leakage into the cytosol.

## 2 RESULTS

### 2.1 **Amelioration of age‐dependent increase in oxidation in the hippocampus of cathepsin B‐deficient (*CatB*** ^−/−^ **) mice**

**Figure 1:**
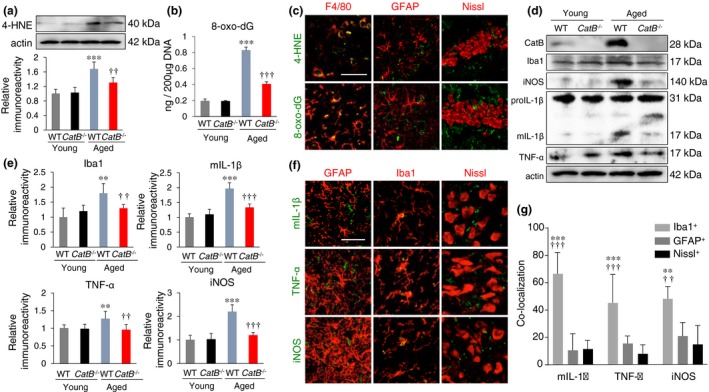
Amelioration of age‐dependent increased oxidation and inflammation in the hippocampus of *CatB* ^−/−^ mice. (a) Immunoblotting and quantitative analyses show HNE in WT and *CatB* ^−/−^ of both young (2 months old) and aged (20 months old) groups. (b) The amount of 8‐oxo‐dG in the hippocampus of WT and *CatB* ^−/−^ of both young and aged groups. (c) CLSM images of the HNE, 8‐oxo‐dG, and merged images with microglia marker F4/80, astrocyte marker GFAP, and neuron marker Nissl in the hippocampus of aged WT mice. Scale bar, 50 μm. (d) Immunoblotting showing CatB, Iba1, iNOS, IL‐1β, and TNF‐α in WT and *CatB* ^−/−^ of both young and aged groups. (e) The quantitative analyses of Iba1, iNOS, mIL‐1β, and TNF‐α in the immunoblots of (d). The results represent the mean ± *SEM* of three independent experiments in a, b, and e. The asterisks indicate a statistically significant difference from the young WT group (**p < 0.01, ***p < 0.001, one‐way ANOVA test). The daggers indicate a statistically significant difference from the aged WT mice (^††^ *p* < 0.01, ^†††^ *p* < 0.001, one‐way ANOVA test). (f) CLSM images of the IL‐1β, TNF‐α, and iNOS with microglia marker Iba1, GFAP, and Nissl in the hippocampus of aged WT mice. Scale bar, 30 μm. (g) The mean percentage of IL‐1β，TNF‐α and iNOS‐positive microglia (Iba1), neurons (Nissl) and astrocytes (GFAP) in (f). Each column and bar represents the means ± *SEM* (*n* = 6, each). Asterisks indicate a statistically significant difference from the value for astrocytes in the same group (**p < 0.01, ***p < 0.01, two‐way ANOVA test), and the daggers indicate a statistically significant difference from the value for neurons in the same group (^††^ *p* < 0.01, ^†††^ *p* < 0.001, one‐way ANOVA test)

We first examined the effects of CatB deficiency on the oxidative stress in the hippocampus during aging. The amounts of 8‐oxo‐7, 8‐dihydro‐2'‐deoxyguanosine (8‐oxo‐dG), and 4‐hydroxynonenal (4‐HNE), which are formed by oxidant interactions with DNA and lipids, respectively, were barely detectable in the hippocampus of young wild‐type (WT) and *CatB* ^−/−^ mice, and there was no significant difference in the mean relative immunoreactivity between these two groups. In contrast, the mean amounts of these oxidative markers were significantly larger in the hippocampus of aged WT mice than in younger animals (Figure 1a,b). The mean relative amount of 8‐oxo‐dG and 4‐HNE was significantly lower in aged *CatB* ^−/−^ mice than in aged WT mice. To identify the possible cellular origin of oxidative stress, double immunohistochemical staining was conducted. In the hippocampus of aged WT mice, the immunoreactivities of both 8‐oxo‐dG and 4‐HNE were found exclusively in microglia with activated morphology, but not in astrocytes or neurons (Figure 1c).

### 2.2 **Amelioration of age‐dependent increase in inflammation in the hippocampus of *CatB*** ^−/−^ **mice**

CatB is involved in maturation of pro‐IL‐1β (Terada et al., 2010) and activation of NF‐κB through the autophagic system (Ni et al., 2015) in microglia. Therefore, the effects of CatB deficiency on the expression of proinflammatory mediators in the hippocampus during aging were further examined.

The mean levels of CatB, inducible nitric oxide synthase (iNOS), mature interleukin‐1β (mIL‐1β) with a molecular mass of 17 kDa, and tumor necrosis factor‐α (TNF‐α) in the hippocampus were significantly higher in aged WT mice, but not in aged *CatB* ^−/−^ mice, than in younger animals (Figure 1d,e). Furthermore, their mean levels in the hippocampus of aged *CatB* ^−/−^ mice were significantly lower than those in aged WT mice (Figure 1d,e). On the other hand, there was no significant age‐dependent change in pro‐IL‐1β with a molecular mass of 31 kDa in either genetic group (Figure 1d). To identify the possible cellular origin of proinflammatory mediators, double immunohistochemical staining was conducted. For immunostaining of IL‐1β, antibody that recognizes only mIL‐1β (17 kDa) was used (m118, Santa Cruz Biotechnology). In the hippocampus of aged WT mice, the immunoreactivity of mIL‐1β, TNF‐α and iNOS was also found exclusively in microglia with activated morphology, but not in astrocytes or neurons (Figure 1f,g).

### 2.3 **Amelioration of age‐dependent decline in learning and memory of *CatB*** ^−/−^ **mice**

**Figure 2:**
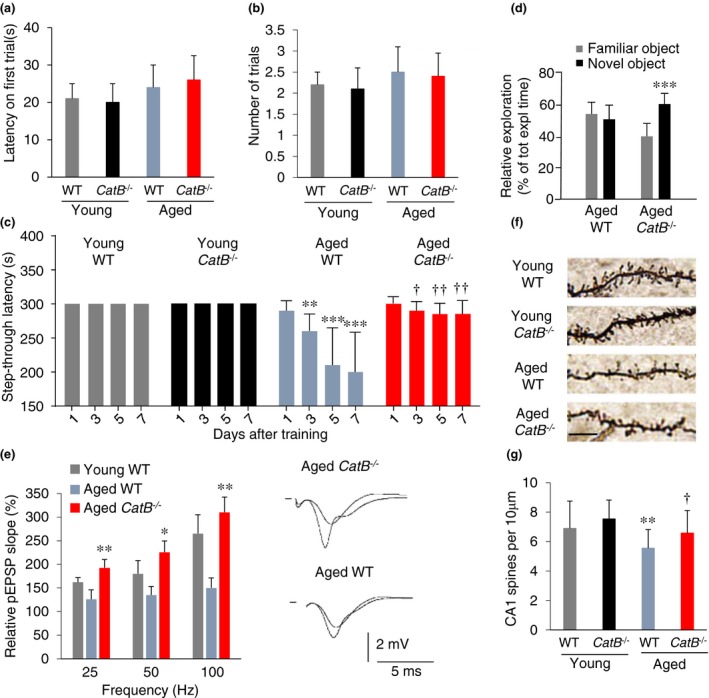
Amelioration of age‐dependent decline in learning and memory in *CatB* ^−/−^ mice. (a) Step‐through latency of WT and *CatB* ^−/−^ mice on the first trial in the acquisition trials. (b) The total number of acquisition trials. (c) Step‐through latencies in the retention trials performed 1, 3, 5, and 7 days after the acquisition trials. The columns and bars represent the mean ± *SEM* of young WT (2 months, *n* = 10), *CatB* ^−/−^ young (2 months, *n* = 10), aged WT (20 months, *n* = 14) and aged *CatB* ^−/−^ (20 months, *n* = 8) mice. (d) Time spent exploring the familiar and the novel object in the recognition trial. The results represent the mean ± *SEM* (aged WT mice, *n* = 6; aged *CatB* ^−/−^ mice, *n* = 6). The asterisks indicate a statistically significant difference from the familiar object (***p < 0.001, Student's *t *test). (e) Cumulative potentiation of fEPSP slope after consecutive tetanic stimulation at 25, 50, and 100 Hz in the hippocampus of aged WT and aged *CatB* ^−/−^ mice. The traces show the typical fEPSPs before and after stimulation at 100 Hz in the hippocampal slices prepared from aged *CatB* ^−/−^ and aged WT mice. The results represent the mean ± *SEM* of five slices from three animals of each group. (f) Representative images of CA1 dendritic branches showing spines of WT and *CatB* ^−/−^ of both young and aged groups. Scale bar, 5 μm. (g) Analysis of spine density in the apical segment of hippocampal CA1 neurons of WT and *CatB* ^−/−^ of both young and aged groups. The results represent the mean ± *SEM* (*n* = 25 dendrites in three mice). The asterisks indicate a statistically significant difference from the young WT group (*p < 0.05, **p < 0.01, and ***p < 0.001, one‐way ANOVA test). The daggers indicate a statistically significant difference from the aged WT group (^†^ *p* < 0.05 and ^††^ *p* < 0.01, one‐way ANOVA test)

It is well known that increased oxidative and inflammatory responses are closely associated with age‐dependent cognitive impairment. The effects of CatB deficiency on the age‐dependent cognitive impairment were therefore examined. Mice entering a dark room during the acquisition trial were used in the subsequent analysis. The latency to escape from the white compartment was defined positively correlated with memory performance, the greater the latency, the better cognition. There was no significant difference in the latency in the first trial or in the number of trials among all groups (Figure 2a,b). The retention latencies of both aged groups were significantly longer than those in the acquisition trial. The retention latency of four consecutive trials was significantly longer in aged *CatB* ^−/−^ mice than in aged WT mice (Figure 2c). The effects of CatB deficiency on the age‐dependent cognitive impairment were further examined using the novel object recognition test, commonly used simple tests for the hippocampus‐dependent learning and memory. Aged WT mice did not show a response and could not discern a change in the object. In contrast, aged *CatB* ^−/−^ mice showed a response to the novel object and were able to discern a change in the object (Figure 2d).

These behavioral observations promoted further examination of the effects of the CatB deficiency on the hippocampal synaptic plasticity. The cumulative potentiation of the field excitatory postsynaptic potential (fEPSP) slope was measured in the Schaffer collateral–CA1 pathway after consecutive tetanic stimulation with 25, 50, and 100 Hz. When the relative fEPSP slope was measured at 30 min after tetanic stimulation, a significant cumulative potentiation was observed in the hippocampus of young WT mice. In contrast, cumulative potentiation was not observed at 30 min even after tetanic stimulation with 100 Hz in the hippocampus of aged WT mice (Figure 2e). On the other hand, the mean values of relative fEPSP slope measured at 30 min after stimulation with 25, 50, and 100 Hz in aged *CatB* ^−/−^ mice were significantly larger than those in aged WT mice (Figure 2e).

We further examined the dendritic spine density of CA1 neurons by Golgi–Cox staining. The mean dendritic spine density of CA1 neurons in the aged *CatB* ^−/−^ mice was significantly larger than that in the aged WT mice (Figure 2f,g).

### 2.4 Involvement of leaking CatB in mitochondria‐derived ROS generation by cultured microglia

**Figure 3:**
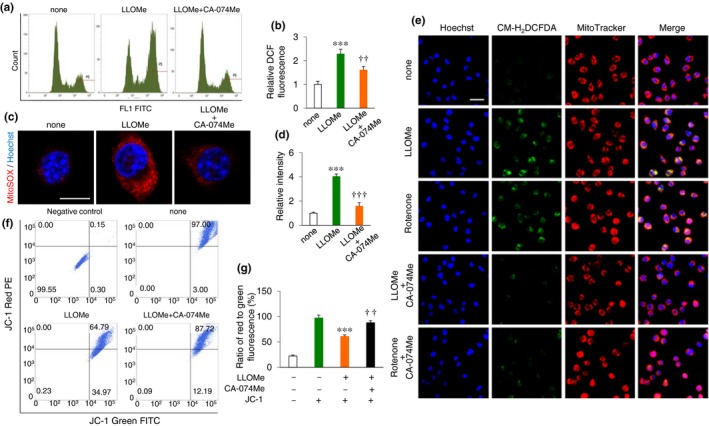
Involvement of leaked CatB in mitochondria‐derived ROS generation and inflammatory response by cultured microglia. (a) Representative FACS histograms of H_2_DCFDA analyses of ROS production in microglia when treated with 100 μM LLOMe or pre‐treated with 50 μM CA‐074Me. (b) The quantitative analyses of the ROS level in the FACS histogram shown in (a). (c) Detection of mitochondria‐derived ROS generated in the cultured microglia using MitoSOX 48 hr after treatment with 100 μM LLOMe or pre‐treatment with 50 μM CA‐074Me. Scale bar, 10 μm. (d) Mean relative intensity of MitoSOX oxidation in the CLSM images of (c). (e) Detection of ROS generated in the microglia using CM‐H_2_DCFDA 48 hr after treatment with 100 μM LLOMe, 100 nM rotenone, or pre‐treatment with 50 μM CA‐074Me. The mitochondria were visualized using MitoTracker. Nuclei were stained by Hoechst. Scale bar, 30 μm. (f) The mitochondrial membrane potential (MMP, Δψm) in microglia was determined by a FACS analysis with JC‐1 staining which reversibly change color from green to red as membrane potential increase. The negative control means cultured cells without JC‐1 staining. The none group means cultured cells without stimulation stained with JC‐1 dye. (g) The quantitative analyses of MG6 cells exhibiting mitochondria‐derived ROS production. The results in b, d, and g represent the mean ± *SEM* of three independent experiments. The asterisks indicate a statistically significant difference from the non‐treated group (***p < 0.001, one‐way ANOVA test). The daggers indicate a statistically significant difference from the LLOMe‐treated group (^††^ *p* < 0.01 and ^†††^ *p* < 0.001, one‐way ANOVA test)

There are some reports showing the increased lysosomal membrane permeability and the resultant leakage of lysosomal enzymes during aging (Nakamura et al., 1989; Nakanishi et al., 1997). Therefore, the effects of lysosomal leakage on the production of ROS were next estimated in microglia by using a flow cytometer and 2’, 7’‐dichlorofluorescein (DCF) diacetate, a fluorescent dye for intracellular hydrogen peroxide. The mean DCF fluorescent intensity in MG6 microglial cell line was significantly increased after treatment with L‐leucyl‐L‐leucine methyl ester (LLOMe), a lysosome‐destabilizing agent that causes lysosomal disruption (Thiele & Lipsky, 1985). As expected, CA‐074Me, a membrane‐permeable selective CatB inhibitor, significantly suppressed the LLOMe‐induced increase in the DCF fluorescent intensity (Figure 3a,b). LLOMe also significantly increased the mean fluorescence signal derived from MitoSOX Red, which emits fluorescence following oxidation by mitochondria‐derived O_2_^−^ generation (Figure 3c,d).

Rather surprisingly, the mean level of MitoSOX Red oxidation in MG6 cells was also significantly inhibited by CA‐074Me (Figure 3c,d). The effects of lysosomal leakage on the ROS generation in MG6 cells were also examined using CM‐H_2_DCFDA, which emits green fluorescence following oxidation by ROS. The green fluorescence signals corresponded closely with those from MitoTracker in MG6 cells treated with LLOMe (Figure 3e), the fluorescence from H_2_DCFDA was significantly inhibited by pre‐treatment with CA‐074Me (Figure 3e). Mitochondria‐derived ROS production is closely linked to a fall in the mitochondrial membrane potential. Therefore, this was confirmed by analyzing the change in the mitochondrial transmembrane electrochemical gradient (Δψm; JC‐1 staining) after lysosomal leakage. The mean Δψm in MG6 cells was significantly decreased after treatment with LLOMe (Figure 3f,g). CA‐074Me also significantly restored the LLOMe‐induced decrease in the mean Δψm (Figure 3f,g).

### 2.5 Increased oxidative stress and inflammation after treatment with rotenone in microglia

To validate the mechanism underlying the lysosomal leakage of CatB and mitochondrial ROS generation, we further examined the possible involvement of CatB in the oxidative and inflammatory responses following treatment with rotenone, which blocks the complex I ubiquinone pathway of the mitochondrial electron transport chain (Palmer, Horgan, Tisdale, Singer, & Beinert, 1968). The unable pass of electron from complex I to ubiquinone created a backup of electrons within the mitochondrial matrix, creating ROS, which can damage DNA and other components of the mitochondria.

**Figure 4:**
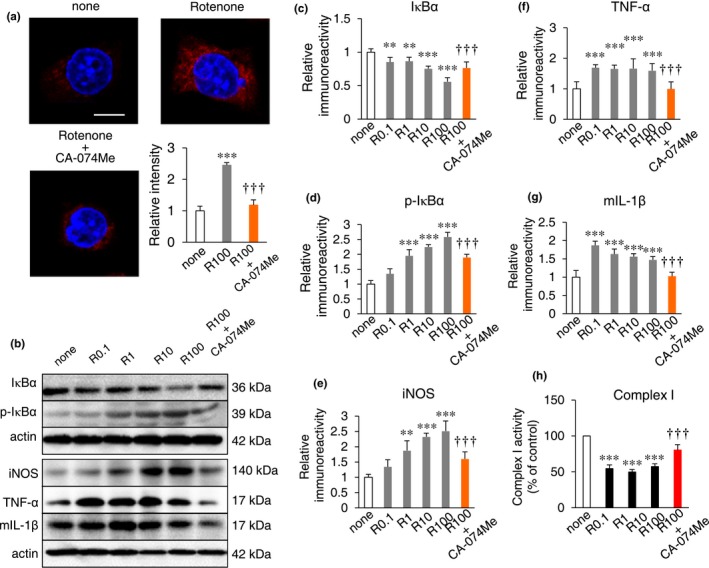
Inflammatory responses after treatment with rotenone in cultured microglia. (a) Detection of mitochondria‐derived ROS generated in the cultured microglia using MitoSOX 48 hr after treatment with 100 nM rotenone or pre‐treatment with 50 µM CA‐074Me. Scale bar, 10 µm. Histograms show the mean relative intensity of MitoSOX oxidation in the CLSM images. (b) Immunoblots showing IκBα, p‐IκBα, iNOS, TNF‐α, and pro‐IL‐1β in the cultured microglia after 48 hr after treatment with 0.1, 1, 10, and 100 nM rotenone or pre‐treatment with 50 µM CA‐074Me. (c, d) The quantitative analyses of IκBα (c) and p‐IκBα (d) in the immunoblots of (b). (e–g) The quantitative analyses of iNOS (e), TNF‐α (f), and mIL‐1β (g) in the Immunoblotting of (b). (h) Detection of the mitochondrial complex I activity in the cultured microglia 48 hr after treatment with 1, 10, and 100 nM rotenone or pre‐treatment with 50 µM CA‐074Me. The results in (a) and (c–h) represent the mean ± *SEM* of three independent experiments. The asterisks indicate a statistically significant difference from the untreated group (**p < 0.01 and ***p < 0.001, one‐way ANOVA test). The daggers indicate a statistically significant difference from the 100 nM rotenone‐treated group (^†††^ *p* < 0.001, one‐way ANOVA test)

Rotenone significantly increased the mean level of MitoSOX Red oxidation and H_2_DCFDA fluorescence in MG6 cells (Figure 4a). Effects of rotenone on the amount of phosphorylated IκBα in MG6 cells were next examined, because phosphorylation of IκBα induces its degradation that is necessary for activation of NF‐κB. Rotenone also significantly decreased the mean amount of IκBα in MG6 cells in a dose‐dependent manner (Figure 4b,c). Furthermore, rotenone significantly increased the mean amounts of p‐IκBα in a dose‐dependent manner (Figure 4b,d). At the same time, rotenone increased the mean amounts of proinflammatory mediators in MG6 cells, including iNOS, TNF‐α, and mIL‐1β (Figure 4b,e–g). At the same time, CA‐074Me significantly suppressed the rotenone‐induced mitochondrial ROS production (Figure 3e), production of proinflammatory mediators, and activation of NF‐κB in MG6 cells (Figure 4b–g). Furthermore, rotenone significantly inhibited the complex I activity in CatB‐dependent manner (Figure 4h).

Either LLOMe or rotenone significantly increased the level of MitoSOX Red oxidation in primary cultured microglia prepared from WT mice (Supporting Information Figure S1). On the other hand, the level of MitoSOX Red oxidation induced by LLOMe or rotenone was markedly declined in primary cultured microglia prepared from *CatB* ^−/−^ mice (Supporting Information Figure S1).

### 2.6 Possible role of CatB leaked into the cytosol in the degradation of pre‐TFAM

**Figure 5:**
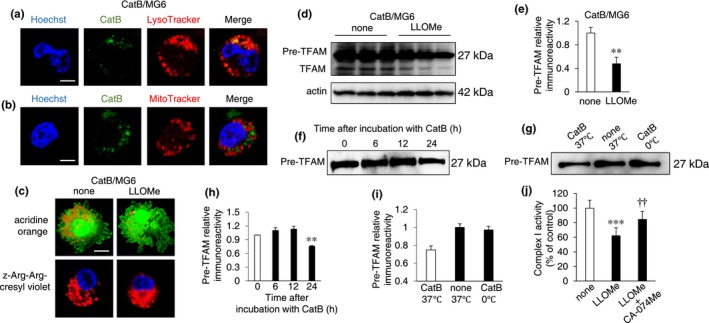
Possible involvement of CatB leaked in the cytosol of microglia in the degradation of TFAM. (a, b) CLSM images of the CatB (green) merged images with LysoTracker (a) and MitoTracker (b) in MG6 cells 48 hr after CatB‐overexpressing plasmid transfection. Scale bar, 5 µm. (c) CLSM images of acridine orange and Z‐Arg‐Arg‐cresyl violet in non‐treated and LLOMe‐treated CatB/MG6 cells. LLOMe quenched fluorescent emission of acridine orange, but not of Z‐Arg‐Arg‐cresyl violet. Scale bar, 5 µm. (d) Immunoblots showing a decrease in TFAM in CatB/MG6 cells pre‐treated with LLOMe. (e) The quantitative analyses of immunoblots in (d). The results represent the mean ± *SEM* of three independent experiments. The asterisks indicate a statistically significant difference from the control (**p < 0.01, one‐way ANOVA test). (f) The immunoblots show degradation of mouse recombinant TFAM by human recombinant CatB at 37°C, pH = 7. (g) The immunoblots show degradation of mouse TFAM 24 hr after incubated with and without CatB at 37°C or 0°C, PH = 7. (h) The quantitative analyses of TFAM in the immunoblotting shown in (f). The results represent the mean ± *SEM* of three independent experiments. The asterisks indicate a statistically significant difference from the control (**p < 0.01, one‐way ANOVA test). (i) The quantitative analyses of TFAM in the immunoblotting shown in (g). (j) Mitochondrial complex I activity in MG6 cells 24 hr after treatment with LLOMe alone or combination of LLOMe with CA‐074Me. The results represent the mean ± *SEM* of three independent experiments. The asterisks indicate a statistically significant difference from the controls (***p < 0.001, one‐way ANOVA test). The daggers indicate a statistically significant difference from the LLOMe alone (^††^ *p* < 0.001, one‐way ANOVA test)

We next examined a possible association of CatB leaked into the cytosol with the degradation of pre‐TFAM. The soluble extract from MG6 cells after transfection with human CatB cDNA (CatB/MG6 cells) showed an increase in the amount of CatB in a transfected cDNA amount–dependent manner (Supporting Information Figure S2a,b). Immunohistochemical staining showed increased granular immunoreactivity of CatB in CatB/MG6 cells (Supporting Information Figure S2c). In the CatB/MG6 cells, the immunoreactivity of CatB corresponded well with LysoTracker (Figure 5a, Supporting Information Figure S3b), but not with MitoTracker (Figure 5b, Supporting Information Figure S3b). We next examined lysosomal destabilization and leakage of CatB in CatB/MG6 cells after treatment with LLOMe using two different reagents. One was an acidity‐dependent acridine orange, and the other was cell‐permeable fluorescently labeled CatB substrate, z‐Arg‐Arg‐cresyl violet, and the fluorescent cresyl violet group of which was designed to be dequenched upon cleavage of one or both of the arginines by CatB. The punctate acridine orange aggregates were observed in non‐treated control CatB/MG6 cells (Figure 5c, Supporting Information Figure S3c). The enzymatic activity of CatB was also visible as punctate bright signals in CatB/MG6 cells. On the other hand, LLOMe markedly reduced the fluorescent signal for acridine orange (Figure 5c). Rather surprisingly, the enzymatic activity of CatB was still remained as diffuse bright signals in CatB/MG6 cells after treatment with LLOMe, suggesting that CatB leaked from lysosomes retained the enzymatic activity even in the cytosol.

To address that full‐length TFAM (pre‐TFAM) as a potential substrate of CatB leaked in the cytosol, the soluble extracts from the non‐treated and LLOMe‐treated CatB/MG6 cells were subjected to immunoblotting using ant‐TFAM antibody. There were two bands corresponding to a 27‐kDa pre‐TFAM, which is localized in the cytosol, and a 24‐kDa mature TFAM, which is localized in the mitochondria (Figure 5d). The mean amount of pre‐TFAM in LLOMe‐treated CatB/MG6 cells was significantly lower than that in non‐treated CatB/MG6 cells (Figure 5d,e). In in vitro digestion assay, human recombinant pre‐TFAM was significantly decreased 24 hr after incubation with CatB in cleavage buffer at neutral pH, 37°C (Figure 5f,g). The amount of human recombinant pre‐TFAM was not decreased when pre‐TFAM was incubation without CatB or incubation at 0°C (Figure 5h,i). Furthermore, the complex I activity was significantly reduced by treatment with LLOMe, whereas LLOMe failed to reduce the complex I activity in the presence of CA‐074Me (Figure 5j).

It has been reported that apoptosis triggered by lysosomal leakage of CatB is mediated by cleavage of Bid in the cytosol (Stoka et al., 2001). The soluble extract from MG6 cells after treatment with LLOMe was evaluated by Western blotting. We detected the 23‐kDa full‐length Bid, but the 15‐kDa t‐Bid (truncated bid) was not visible even after LLOMe treatment in MG6 cells (Supporting Information Figure S3a).

### 2.7 Impairment of learning and memory in middle‐aged mice by intralateral ventricle injection of CatB/MG6 cells

**Figure 6:**
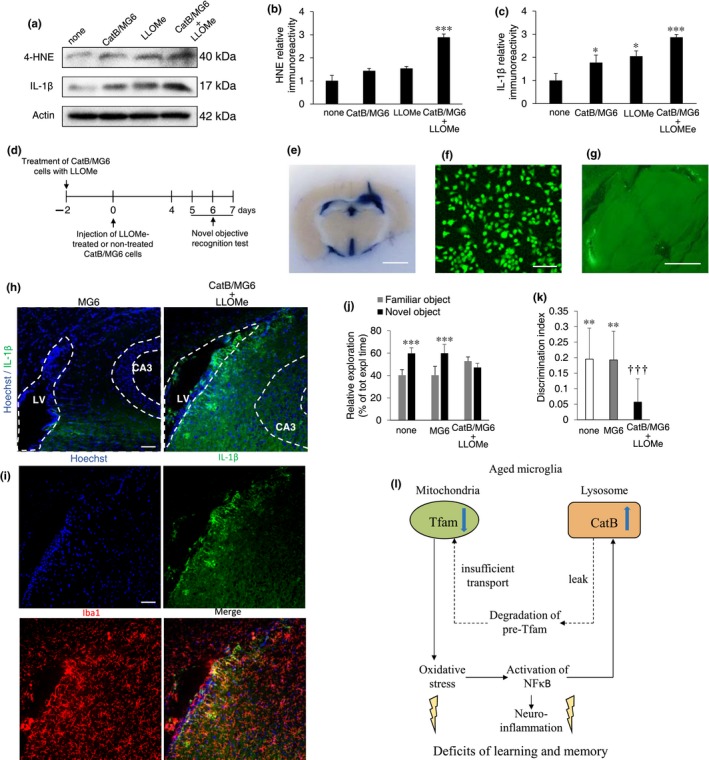
Impairment of learning and memory in middle‐aged mice by intralateral ventricle injection of CatB‐overexpressing microglia. (a) Immunoblots showing HNE and IL‐1β 48 hr after CatB‐overexpressing plasmid transfection, LLOMe treatment in MG6 cells, and LLOMe treatment in CatB/MG6 cells. (b, c) The quantitative analyses of HNE and IL‐1β in the immunoblotting shown in (a). The results represent the mean ± *SEM* of three independent experiments. The asterisks indicate a statistically significant difference from the controls (*p < 0.05, ***p < 0.001, one‐way ANOVA test). (d) Experimental timeline. (e) Evens blue was injected into the right lateral ventricle, and the dye was distributed throughout the mouse ventricular system. Scale bar, 2 mm. (f) CLSM images of MG6 cells labeling by CFDA SE cell tracer. Scale bar, 50 µm. (g) CLSM image of transplanted MG6 cells in lateral ventricle 3 days after transplantation. Scale bar, 2 mm. (h) CLSM images of Hoechst and mIL‐1β in the periventricular area and hippocampal CA3 subfield of middle‐aged mice after intralateral ventricular injection of MG6 and LLOMe‐treated CatB/MG6. Scale bar, 100 µm. (i) CLSM images of Hoechst, mIL‐1β, and Iba1 in the periventricular area and hippocampal CA3 subfield after intralateral ventricular injection of LLOMe‐treated CatB/MG6. Scale bar, 100 µm. (j, k) The recognition memory was evaluated by the novel object recognition test in 10‐month‐old mice 5 days after intralateral ventricle injection of cultured medium (control), MG6 cells, and LLOMe‐treated CatB/MG6 cells. (j) Time spent exploring the familiar and the novel object in the recognition trial. The results represent the mean ± *SEM* (cultured medium injection [control], *n* = 15; MG6 cell transplantation [MG6], *n* = 14; LLOMe‐treated CatB/MG6 cell transplantation [CatB/MG6 + LLOMe], *n* = 12). The asterisks indicate a statistically significant difference from the vehicle group (***p < 0.001, Student's *t *test). (k) Discrimination index (exploration of novel object minus exploration of familiar object/total exploration time). The results represent the mean ± *SEM* (cultured medium injection [control], *n* = 15; MG6 cells transplantation [MG6], *n* = 14; LLOMe‐treated CatB/MG6 cells transplantation [CatB/MG6 + LLOMe], *n* = 12). The asterisks indicate a statistically significant difference from 0 (**p < 0.01 and ***p < 0.001, Student's *t* test). The daggers indicate a statistically significant difference from the MG6 transplantation group (^†††^ *p* < 0.001, one‐way ANOVA test). (l) A schematic illustration representing the CatB leaked in the cytosol during normal aging play a critical role in the mitochondria‐derived ROS generation and inflammatory response through proteolytic degradation of TFAM, resulting in impairment of learning and memory

Next, we examined the effect of intralateral ventricle injection of LLOMe‐treated CatB/MG6 cells, which mimic aged microglia, in middle‐aged mice on the cognitive function. There was no significant difference in the mean immunoreactivity of 4‐HNE between MG6 and MG6/CatB cells. However, the mean immunoreactivity of 4‐HNE was significantly increased after treatment with LLOMe in CatB/MG6 cells, but not in MG6 cells (Figure 6a,b). In contrast, the mean immunoreactivity of mIL‐1β was significantly greater in CatB/MG6 cells than in MG6 cells (Figure 6a,c). Furthermore, the mean immunoreactivity of mIL‐1β in CatB/MG6 cells was further increased after treatment with LLOMe (Figure 6a,c).

The effects of intralateral ventricle injection of culture medium (control), MG6 cells, and LLOMe‐treated CatB/MG6 cells on the recognition memory of middle‐aged mice were examined (Figure 6d). Successful injection into the lateral ventricles was confirmed using Evans Blue (Figure 6e) and CFDA‐stained MG6 cells (Figure 6f,g). At the site of injection, large double‐positive cells for Iba1 and mIL‐1β that were considered to be injected LLOMe‐treated CatB/MG6 cells attached to ependymal cells along the lateral ventricle extended their processes to the stratum oriens of the hippocampal CA3 subfield (Figure 6h,i). Furthermore, some large double‐positive cells for Iba1 and mIL‐1β localized in the hippocampal CA3 subfield. Infiltrated LLOMe‐treated CatB/MG6 cells may further activate the surrounding brain resident microglia through secretion of mIL‐1β, because microglia with smaller cell bodies also expressed mIL‐1β. In contrast, immunoreactivity for mIL‐1β was not detected in either injected site or the hippocampal CA3 subfield after injection of MG6 cells (Figure 6h,i).

There was no significant difference in the mean latency or the mean total exploration time in any of three groups (Supporting Information Figure S4). However, while the middle‐aged mice subjected to the intralateral ventricle injection of culture medium or MG6 cells showed a response to the novel object and were able to discern a change in the object, the middle‐aged mice subjected to the intralateral ventricle injection of LLOMe‐treated CatB/MG6 cells did not show a response and could not discern a change in the object (Figure 6j,k).

## 3 DISCUSSION

CatB increased in the hippocampal microglia during aging is responsible for age‐dependent increase in oxidative stress, inflammatory responses, and impairment of learning and memory. A leakage of CatB into the cytosol may trigger these responses, as there are some reports showing the increased lysosomal membrane permeability and the resultant leakage of lysosomal enzymes during aging (Nakamura et al., 1989; Nakanishi et al., 1997). Furthermore, the immunoreactivity for CatB was markedly increased in the hippocampal microglia of mice during aging (Wu et al., 2017). It is important to note that resident microglia are long‐lived cells that can survive the entire mouse lifespan (Füger et al., 2017). These observations suggest that the fragility of the endosomal/lysosomal system of especially long‐lived microglia is markedly increased during aging. Furthermore, the lysosomal membrane is protected from acidic hydrolases by the lysosome‐specific expression of membrane proteins, such as lysosomal‐associated membrane protein (LAMP) 1 and LAMP2, which are heavily glycosylated and hence resist digestion (Eskelinen, 2006). Therefore, the age‐dependent decrease in the gene expression of these lysosomal membrane proteins is also involved in an age‐dependent increase in the lysosomal membrane permeabilization (Huang, Xu, Pang, Bai, & Yan, 2012).

Present observations also suggest that lysosomal leakage of CatB plays a critical role in a fall in the mitochondrial membrane potential, leading to mitochondria‐derived ROS generation. After leakage, CatB is known to process the full‐length Bid to t‐Bit, which is essential for permeabilization of the outer mitochondria membrane (Stoka et al., 2001). In the present study, however, t‐Bid was not detected in the soluble extract from MG6 cells after treatment with LLOMe, indicating that the lysosomal leakage of CatB and the resultant generation of ROS were not associated with apoptotic process of microglia. Therefore, it is considered that CatB may cleave cytosolic substrates rather than Bid, resulting in an increase in oxidative stress and inflammatory responses.

Transcription factor A is synthesized as a full‐length precursor (pre‐TFAM) in the cytosol and then transported into the mitochondrial matrix (Nourshahi, Damirchi, Babaei, Gholamali, & Salehpour, 2012). Besides the maintenance of the mtDNA number as a transcription factor, TFAM can stabilize mtDNA through the formation of a nucleoid structure within the mitochondria (Kanki et al., 2004). To stabilize a ratio of TFAM and mtDNA, excess TFAM is selectively degraded by Lon, the major protease in the mitochondria matrix (Matsushima et al., 2010). Therefore, TFAM synthesized in the cytosol is a potential target substrate for CatB leaked in the cytosol of microglia during aging. In the present study, the mean amount of pre‐TFAM in CatB‐overexpressing MG6 (MG6/CatB) cells after treatment with LLOMe was significantly lower than that in non‐treated cells. Furthermore, CatB can degrade human recombinant TFAM even under the neutral pH. Given these results, we may conclude that CatB leaked in the cytosol during aging induces the mitochondrial disruption through degradation of TFAM. Therefore, subnormal level of TFAM in microglia may induce a decline of the complex I activity. The decreased complex I activity then induces an increase in the mitochondria‐derived ROS generation and inflammatory responses.

In the present study, rotenone also increased the mean amounts of p‐IκBα and proinflammatory mediators. Rotenone, which binds at or near the ubiquinone binding site, does not directly inhibit the complex I activity (Palmer et al., 1968). Therefore, observations here suggest that the inhibitory effects of rotenone on the complex I activity may be mediated by lysosomal leakage of CatB, as rotenone effectively triggers mitochondria‐derived ROS generation by increasing the lysosomal membrane permeability (Wu et al., 2015). As expected, CA‐074Me significantly suppressed the rotenone‐induced increase in the mean amounts of p‐IκBα and proinflammatory mediators. Furthermore, CA‐074Me also significantly suppressed the rotenone‐induced inhibition of complex I activity.

The role of CatB in the cognitive function remains controversial. Pharmacological or genetic inhibition of CatB decreases Aβ levels and improves the memory function in mouse models of Alzheimer's disease (Hook, Kindy, Reinheckel, Peters, & Hook, 2009; Kindy et al., 2012). However, Embury et al. (2017) have conversely reported that the overexpression of CatB in hippocampal neurons using adeno‐associated virus serotype 2/1 ameliorates Alzheimer's disease‐like pathologies, including β‐amyloidosis and impairments in learning and memory in the mouse brain. Furthermore, Moon et al. (2016) reported that CatB has beneficial effects on cognition, such as enhanced hippocampal neurogenesis and spatial memory in mice following exercise. One possible explanation for these discrepancies in findings regarding the role of CatB in the cognitive function is the functions of CatB differ among cell types.

During aging, increased activation of NF‐κB may enhance the expression of CatB, especially in microglia, as its promoter region possesses the NF‐κB binding site (Ni et al., 2015). Increased CatB further activates NF‐κB through the autophagic system in microglia (Ni et al., 2015). Therefore, genetic depletion of CatB may have much more influence on microglial functions rather than neuronal functions during aging, because *CatB* ^−/−^ mice show no detectable phenotypes including memory ability until their middle age (Wu et al., 2017). This may also suggest that the developmental issue may be not involved in the present findings in aged *CatB* ^−/−^ mice. Studies using the mice with microglia‐specific depletion of CatB will be needed to further clarify this issue.

In conclusion, our observations demonstrate that increase and leakage of CatB in microglia during aging are responsible for the increased generation of mitochondria‐derived ROS and proinflammatory mediators, culminating in memory impairment (Figure 6l).

## 4 EXPERIMENTAL PROCEDURES

### 4.1 Mice

Heterozygous mice of C57BL/6N background were kept under a specific pathogen‐free condition at Kyushu University Faculty of Dental Sciences. Selection of *CatB* ^−/−^ mice was performed according to the method described previously (Terada et al., 2010). Young (2 months old) and aged (20 months old) of both C57BL/6N WT and *CatB* ^−/−^ male mice and middle‐aged C57BL/6N WT male mice (10 months old) were used in the present study. The WT mice were derived from the same breeding colony. All experimental procedures of this study were approved by the Animal Care and Use Committee of Kyushu University.

### 4.2 Immunoblotting analyses

The hippocampus for immunoblotting analyses was prepared as described previously (Ni et al., 2015). Primary antibodies were incubated overnight at 4°C. Second antibodies were incubated 1 hr at room temperature. The HRP‐labeled antibodies were detected using an ECL kit with an image analyzer (LAS‐1000; Fuji Photo Film).

### 4.3 8‐oxo‐dG assay

The hippocampus samples prepared from each group were cut up into small pieces and subjected to DNA extraction by DNA Extractor TIS Kit. The extracted DNA were prepared using 8‐oxo‐dG Assay Preparation Reagent Set and analyzed using an 8‐oxo‐dG ELISA kit in accordance with the manufacturer's protocol.

### 4.4 Immunofluorescent staining

Mice brain samples for immunofluorescent staining were performed as previously reported (Wu et al., 2013). The fluorescence images were observed using a confocal laser scanning microscope (CLSM; C2si, Nikon). For the MG6 cell staining, the cells transfected with CatB‐overexpressing plasmid or combination of LLOMe (24 hr after transfection) were incubated with 500 nM MitoTracker Red or 50 nM LysoTracker Red DND‐99 for 30 min at 37°C, and then subjected to immunostaining for CatB.

### 4.5 Electrophysiology

Hippocampal sagittal slices (400 µm thick) prepared from young WT mice and aged WT and *CatB* ^−/−^ mice were used to measure the cumulative potentiation of the fEPSP slope evoked in the Schaffer collateral–CA1 pathway as described previously (Hayashi et al., 2008).

### 4.6 Golgi–Cox staining

The brains were prepared and stained in accordance with manufacturer's instructions (Super Golgi Kit). All images were processed using the ImageJ software program (National Institute of Health, Bethesda, MD). The spine density was determined by manually identifying the spines and using ImageJ to measure the dendrite length.

### 4.7 Cell culture

The mouse microglial cell line MG6 (Riken BioResource Center: Cell No. RCB2403) was maintained in accordance with the previously described methods (Ni et al., 2015). The primary microglia from WT and *CatB* ^−/−^ mice were prepared from the neonatal cortex in accordance with the previously described methods (Ni et al., 2015).

### 4.8 Flow cytometry

The MG6 cells were treated with 100 μM LLOMe or 1 hr pre‐treated CA‐074Me. After 48 hr, the cells were harvested and suspended in HBSS with 10 μmol/L of CM‐H2DCFDA for 45‐min incubation. A total of 5,000 events were analyzed using flow cytometry (BD FACSVerse). For mitochondrial membrane potential analyses, a JC‐1 assay was conducted. Cells were labeled with 2 μM JC‐1 at 37°C, 5% CO_2_ for 30 min. FACS analyses were conducted using 488‐nm excitation with 530/30 nm and 585/42 nm bandpass emission filters.

### 4.9 Detection of mitochondrial ROS

ROS production by MG6 cells was detected using MitoSOX Red and DCFA as described previously (Ni et al., 2015).

### 4.10 Fluorescence imaging of lysosomes and CatB

CatB/MG6 cells were stained with the cell‐permeable fluorescently labeled CatB substrate Z‐Arg‐Arg‐cresyl violet or with Hoechst stain with 5 mM acridine orange according to the manufacturer's instruction. The live cells were then observed using a CLSM (C2si; Nikon).

### 4.11 In vitro digestion assay

Mouse recombinant TFAM (0.25 μg) was incubated with 0.2 μg human recombinant CatB in 20 μl cleavage buffer (25 mM HEPES, 1 mM EDTA, 0.1% CHAPS, 10 mM DTT, 10% sucrose, PH = 7) at 37°C for 0, 6, 12, and 24 hr. The mixture at 37°C without CatB and mixture at 0°C with CatB were set as negative control. The resulting reaction mixtures were analyzed by western blotting.

### 4.12 Transplantation

Middle‐aged mice were subjected to MG6 transplantation under anesthesia. To visualize transplanted cells, CatB/MG6 cells and MG6 cells were incubated with CFDA (Invitrogen) for 15 min at 37°C. Cells were re‐pelleted and suspended in DMEM at a final concentration of 1 × 10^6^ cells in 5 μl and then transplanted to the right lateral ventricle from the bregma (−0.55 mm posterior, −1.1 mm lateral and 2.5 mm ventral). After operation, mice were returned to the cage.

### 4.13 Mice behavioral studies

Young WT, *CatB* ^−/−^ young, aged WT and aged *CatB* ^−/−^ mice were used to examine the learning and memory by step‐through avoidance test and novel object recognition test. Novel object recognition test was also used to examine the learning and memory of middle‐aged mice in cultured medium injection group, MG6 cell transplantation group and LLOMe‐treated CatB/MG6 cell transplantation group.

### 4.14 Statistical analysis

The data are represented as the means ± *SEM*. The statistical analyses were performed by a one‐ or two‐way ANOVA with a post hoc Tukey's test using the GraphPad Prism software package (GraphPad Prism 7). A value of *p* < 0.05 was considered to indicate statistical significance.

Additional experimental procedures are available in Supporting Information.

## CONFLICT OF INTEREST

None declared.

## AUTHOR CONTRIBUTIONS

J.N. conducted most of the experiments, analyzed the data, and wrote the manuscript. Z.W. designed and conducted some histological experiments and wrote the manuscript. V.S. analyzed the data. J.M. and Y.H. performed electrophysiological experiments. C.P. and T.V. provided advice about data interpretation. H.Q. provided unpublished reagents/analytic tools. H.N. designed the whole study and wrote the manuscript.
