# Supplementary material for: Increased expression and altered subcellular distribution of cathepsin B in microglia induce cognitive impairment through oxidative stress and inflammatory response in mice
Source: Aging Cell. 2018 Dec 21;18(1):e12856. doi: 10.1111/acel.12856 (PMC6351837; doi:10.1111/acel.12856)

**SUPPLEMENTAL INFORMATION**

**Increased expression and altered subcellular distribution of cathepsin B in microglia induces cognitive impairment through oxidative stress and inflammatory response in mice**

Junjun Ni,^1^ Zhou Wu,^1^ Veronika Stoka,^2^ Jie Meng,^1^ Yoshinori Hayashi,^1^ Christoph Peters,^3^

Hong Qing, ^4^ Vito Turk, ^2^ Hiroshi Nakanishi^1, 5^

**Supplemental figure legends**

**Figure S1.** **Detection of mitochondria-derived ROS generated in the cultured primary microglia.** ROS generation originated from the mitochondria was detected in primary cultured microglia prepared from WT and *CatB^-/-^* mice using MitoSOX 48 h after treatment with 100 μM LLOMe or 100 nM rotenone. Scale bar, 10 μm.

**Figure S2.** **Overexpression of CatB in MG6 cells.** (A) Immunoblots showing CatB 48 h after 0.1 μg, 0.5 μg and 1 μg CatB-overexpressing plasmid transfection into 6-well-plated MG6 cells. (B) The quantitative analyses of CatB in the immunoblotting shown in (A). The results represent the mean ± SEM of three independent experiments. The asterisks indicate a statistically significant difference from the control (***P*<0.01, ****P*<0.001, one-way ANOVA test). (C) Immunofluorescent CLSM images of the CatB (green) with a Hoechst stained nucleus in cultured microglia 48 h after 0.5 μg CatB plasmids transfection. Scale bar, 20 μm.

**Figure S3. A possible cleavage of Bid by the leaked CatB from lysosome in MG6 cells after treatment with LLOMe.** (A) The immunoblots show that the full length of Bid (23 kDa) didn’t change after LLOMe treatment and that tBid (15 kDa) wasn’t observed in the cells after LLOMe treatment. (B) Immunofluorescent CLSM images of the CatB (green) merged images with LysoTracker (red, A) and MitoTracker (red, B) in MG6 cells 48 h after CatB- overexpressing plasmid transfection. Scale bar, 20 μm. (C) Fluorescent images of acridine orange and Z-Arg-Arg-cresyl violet in non-treated, LLOMe and CA-074Me-treated CatB/MG6 cells. CA-074Me-treated CatB/MG6 showing a dim CatB enzymatic activity indicated the specificity of Z-Arg-Arg-cresyl violet for CatB. Scale bar, 20 μm.

**Figure S4.** The mean latency and the mean total exploration time for the novel object recognition tests in mice subjected to intra-lateral ventricular injection of MG6 and LLOMe-treated CatB/MG6 cells. The mean latency of first approach to the novel object (A) and the mean total exploration time (B) for the recognition memory in 10 month old mice 5 days after intra-lateral ventricle injection of cultured medium (control), MG6 cells and LLOMe-treated CatB/MG6 cells.

**Supplemental experimental procedures**

**Plasmid construction and transfection of the CatB gene**

The 1.02-kb human CatB EcoRI-EcoRV fragment was cloned from the cDNA library from human THP1 cell line by PCR primer (forward: 5’-CCGGAATTCACCATGTGG CAGCTCT-3’; reverse: 5’-CCGGATATCTTAGATCTTTTCCCAGT-3’). The PCR product was then cloned into pcDNA3.1. In accordance with the Lipofectamin2000 protocol, the MG6 cells were plated at a density of 2.5×10^5^/ml. The DNA and Lipofectamine2000 were diluted in OPTI-MEM and incubated at 24°C for 5 min. The mixture was added to the cell cultured in FBS-free DMEM. The medium was changed to fresh DMEM containing FBS after 6 h.

**Step-through avoidance test**

Step-through avoidance test was performed in identical compartments consisting of illuminated (9×11.5×15 cm) and dark (9×11.5×15 cm) compartments with a grid floor and a guillotine door separating the compartments (Passive avoidance system; Muromachi Kikai Co. Ltd., Tokyo, Japan). Passive avoidance is fear-motivated tests classically used to assess short-term or long-term memory in which the memory performance is positively correlated with the latency to escape from the white compartment. During the training trials, a mouse was placed in the lighted compartment of the box. When the mouse entered the dark compartment, a shock was delivered via the grid floor. The mouse was then removed from the apparatus 30 s after receiving the shock. The same procedure without any foot shock was repeated after an interval of 24 h to assess the level of retention.

**Novel object recognition test**

Mice were individually habituated to an open-field box (58×42×35 cm) by being given 10 min of exploration time in the box without any objects present for 3 d (habituation session). During the acquisition phase, 2 objects of the same material were placed in symmetrical positions in the center of the box for 10 min. One hour after the acquisition phase training, one of the objects was replaced by a novel object, and the exploratory behavior was again analyzed for 3 min. After each session, the objects were thoroughly cleaned with 75% ethanol to prevent odor recognition. Exploration of an object was defined as rearing on the object or sniffing it at a distance of < 1 cm, touching it with the nose, or both. Successful recognition of a previously explored object was reflected by preferential exploration of the novel object. Discrimination of spatial novelty was assessed by comparing the difference between the time of exploration of the novel and familiar object and the total time spent exploring both objects, which made it possible to adjust for differences in total exploration time.

**Resources table**

| REAGENT or RESOURCE | SOURCE | IDENTIFIER |
| --- | --- | --- |
| Antibodies | | |
| Goat anti-CatB | Santa Cruz Biotechnology | Cat#sc-6493 |
| Rabbit anti-4-HNE | Alpha Diagnistic | Cat#HNE11-S |
| Mouse anti-iNOS | Abcam | Cat#ab129372 |
| Mouse anti-IL-1β | Santa Cruz Biotechnology | Cat#sc-74135 |
| Goat anti-cleaved IL-1β (m118) | Santa Cruz Biotechnology | Cat#sc-23460 |
| Rat anti-Bid | R&D | Cat#MAB860 |
| Mouse anti-TNF-α | Abcam | Cat#ab1793 |
| Rabbit anti-TFAM | Calbiochem | Cat#DR1071 |
| Mouse anti-p-IκBα | Santa Cruz Biotechnology | Cat#sc-8404 |
| Rabbit anti-IκBα | Santa Cruz Biotechnology | Cat#sc-847 |
| Mouse anti-actin | Abcam | Cat#ab49900 |
| Goat IgG Horseradish Peroxidase-conjugated Antibody | R&D | Cat#HAF109 |
| ECL Anti-mouse IgG, Horseradish Peroxidase linked whole Antibody | GE Healthcare | Cat#NA931 |
| ECL Anti-rat IgG, Horseradish Peroxidase linked whole Antibody | GE Healthcare | Cat#NA935 |
| ECL Anti-rabbit IgG, Horseradish Peroxidase linked whole Antibody | GE Healthcare | Cat#NA934 |
| Rat anti-F4/80 | Abcam | Cat#ab6640 |
| Rabbit anti-GFAP | Abcam | Cat#ab7260 |
| Anti-8-oxo-dG Monoclonal Antibody | NOF Corporation | Cat#N213120 |
| rabbit anti-Iba1 | Wako | Cat#019-19741 |
| Alexa Fluor® 488 AffiniPure Donkey Anti-Goat IgG (H+L) | Jackson ImmunoResearch | Cat#705-545-003 |
| Cy™3 AffiniPure Donkey Anti-Mouse IgG (H+L) | Jackson ImmunoResearch | Cat#711-165-152 |
| Cy™3 AffiniPure Donkey Anti-Mouse IgG (H+L) | Jackson ImmunoResearch | Cat# 711-165-150 |
| Alexa Fluor® 488 AffiniPure Donkey Anti-Mouse IgG (H+L) | Jackson ImmunoResearch | Cat#715-545-150 |
| Chemicals and Recombinant Proteins | | |
| Sudan Black B | Sigma-Aldrich | Cat#199664 |
| L-Leucyl-L-Leucine methyl ester | Cayman | Cat#6491-83-4 |
| Rotenone | Sigma-Aldrich | Cat#R8875 |
| Hoechst | Sigma-Aldrich | Cat#14533 |
| CA-074Me | PEPTIDE | Cat#4323-v |
| Mouse recombinant TFAM | Cloud-Clone Corp | Cat#RPH050Mu01 |
| Human recombinant CatB | R&D | Cat#953-CY |
| Critical Commercial Assays | | |
| DNA extraction by DNA Extractor TIS Kit | Wako | Cat#296-67701 |
| 8-oxo-dG Assay Preparation Reagent Set | Wako | Cat#292-67801 |
| High Sensitive 8-oxo-dG Check | JaICA | Cat#KOG-HS10E |
| NeuroTrace Fluorescent Nissl Stains | Invitrogen | Cat#N21482 |
| MitoTracker Red | Invitrogen | Cat#M22426 |
| LysoTracker Red DND-99 | Invitrogen | Cat#L7528 |
| Super Golgi Kit | Bioenno Tech | Cat#003010 |
| CM-H_2_DCFA | Invitrogen | Cat#C6827 |
| MitoSOX Red | Invitrogen | Cat#M36008 |
| CV-Cathepsin B Detection Kit | Enzo | Cat#AK-125 |
| Vybrant CFDA SE Cell Tracer Kit | Invitrogen | Cat#V12883 |
| Immobilon ECL Western HRP Substrate | Merck | Cat#WBULS0100 |
| MitoProbe JC-1 Assay Kit | Molecular Probes | Cat#M34152 |
| Software | | |
| GraphPad Prism 7 | GraphPad | https://graphpad.com/scientific-software/prism/ |
| Fiji-ImageJ | National Inst. Of Health | https://imagej.net/fiji |

**Figure S1**


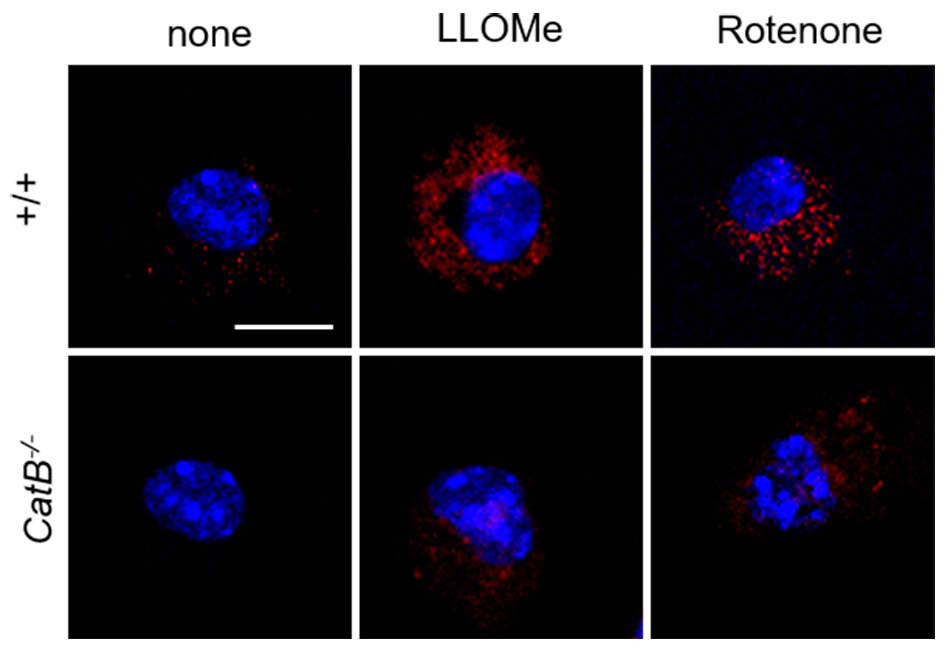


**Figure S2**


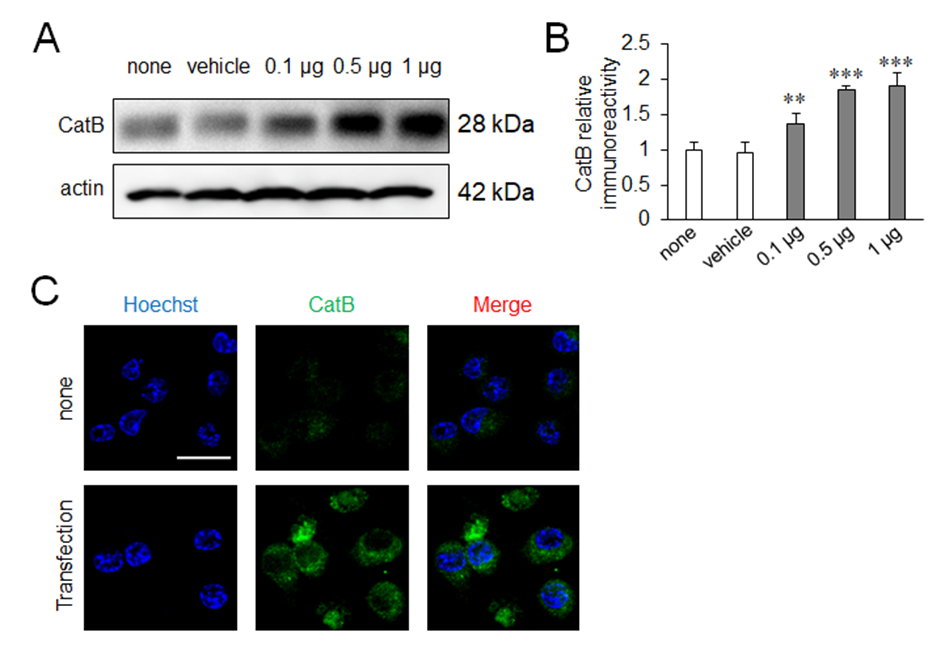


**Figure S3**


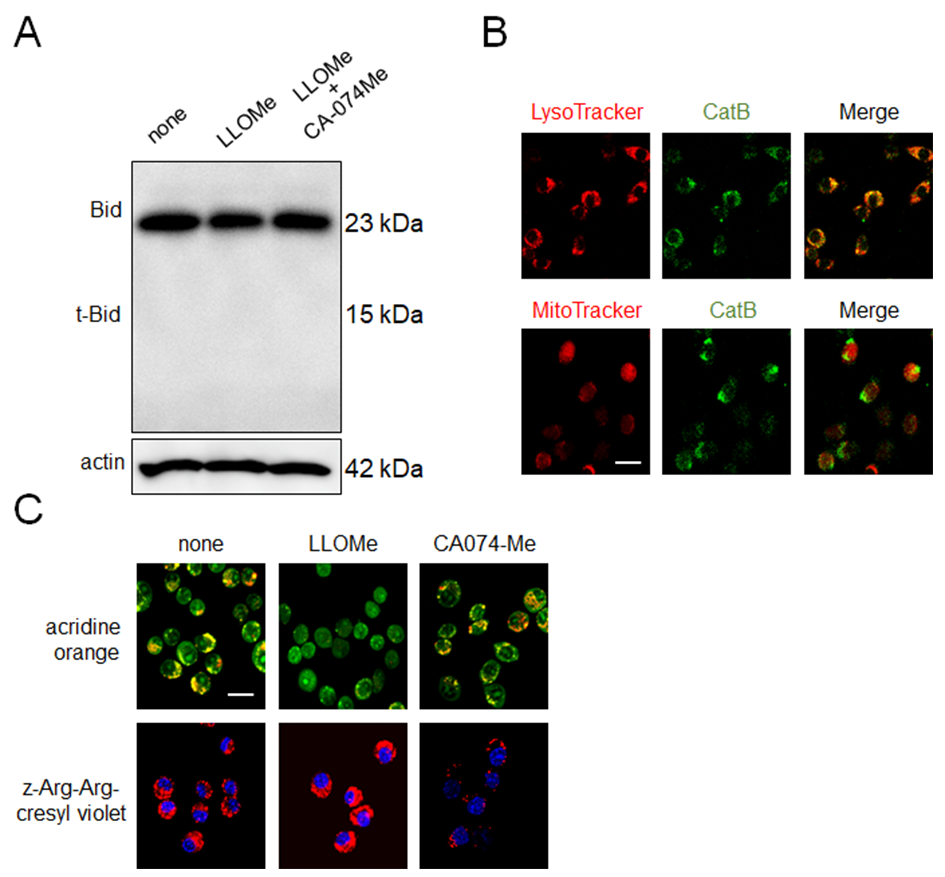


**Figure S4**


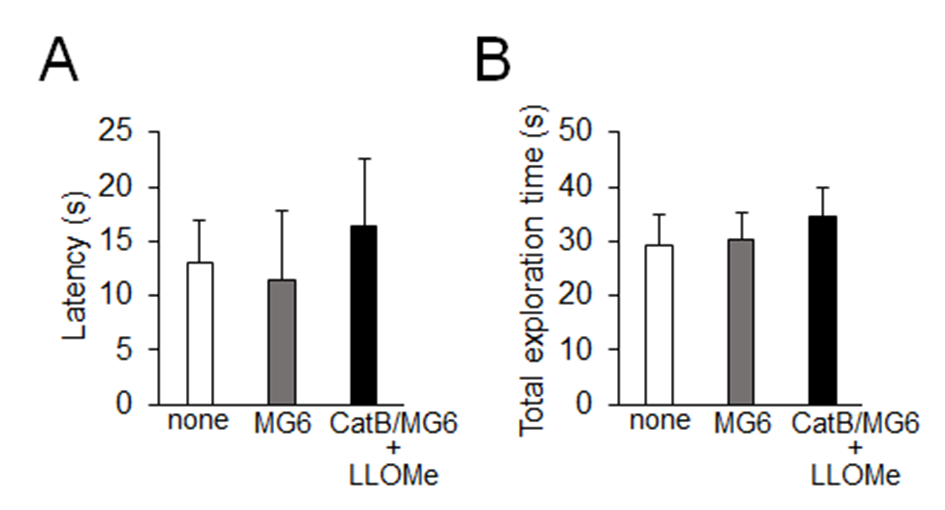

Supplement: Supplementary file 1 [file ACEL-18-e12856-s001.docx]
